# Deep Learning on Oral Squamous Cell Carcinoma Ex Vivo Fluorescent Confocal Microscopy Data: A Feasibility Study

**DOI:** 10.3390/jcm10225326

**Published:** 2021-11-16

**Authors:** Veronika Shavlokhova, Sameena Sandhu, Christa Flechtenmacher, Istvan Koveshazi, Florian Neumeier, Víctor Padrón-Laso, Žan Jonke, Babak Saravi, Michael Vollmer, Andreas Vollmer, Jürgen Hoffmann, Michael Engel, Oliver Ristow, Christian Freudlsperger

**Affiliations:** 1 Department of Oral and Maxillofacial Surgery, University Hospital Heidelberg, 69120 Heidelberg, Germany; Sameena.Sandhu@med.uni-heidelberg.de (S.S.); Michael.Vollmer@med.uni-heidelberg.de (M.V.); andr.vollmer@gmail.com (A.V.); Juergen.Hoffmann@med.uni-heidelberg.de (J.H.); Michael.Engel@med.uni-heidelberg.de (M.E.); oliver.ristow@med.uni-heidelberg.de (O.R.); christian.freudlsperger@med.uni-heidelberg.de (C.F.); 2 Department of Pathology, University Hospital Heidelberg, 69120 Heidelberg, Germany; Christa.Flechtenmacher@med.uni-heidelberg.de; 3 M3i GmbH, 80336 Munich, Germany; ik@m3i-muenchen.de (I.K.); fn@m3i-muenchen.de (F.N.); 4 Munich Innovation Labs GmbH, 80336 Munich, Germany; vpl@munich-innovation.com (V.P.-L.); zj@munich-innovation.com (Ž.J.); 5 Department of Orthopedics and Trauma Surgery, Medical Centre-Albert-Ludwigs-University of Freiburg, Faculty of Medicine, Albert-Ludwigs-University of Freiburg, 79106 Freiburg, Germany; Babak.Saravi@hotmail.de

**Keywords:** confocal microscopy, deep learning, CNN, OSCC, SCC, intraoperative microscopy, AI, ex vivo FCM

## Abstract

Background: Ex vivo fluorescent confocal microscopy (FCM) is a novel and effective method for a fast-automatized histological tissue examination. In contrast, conventional diagnostic methods are primarily based on the skills of the histopathologist. In this study, we investigated the potential of convolutional neural networks (CNNs) for automatized classification of oral squamous cell carcinoma via ex vivo FCM imaging for the first time. Material and Methods: Tissue samples from 20 patients were collected, scanned with an ex vivo confocal microscope immediately after resection, and investigated histopathologically. A CNN architecture (MobileNet) was trained and tested for accuracy. Results: The model achieved a sensitivity of 0.47 and specificity of 0.96 in the automated classification of cancerous tissue in our study. Conclusion: In this preliminary work, we trained a CNN model on a limited number of ex vivo FCM images and obtained promising results in the automated classification of cancerous tissue. Further studies using large sample sizes are warranted to introduce this technology into clinics.

## 1. Introduction

According to global cancer statistics, oropharyngeal cancer is amongst the leading causes of cancer deaths worldwide, particularly in men. Incidence rates vary depending on the presence of risk factors, such as tobacco, alcohol, or chewing of betelnuts combined with poor oral hygiene and limited access to medical facilities. In Europe, head and neck cancers represent 4% of all malignancies. The most common entity among them is oral squamous cell carcinoma (OSCC), which can be found in more than 90% of patients with head and neck cancer [1]. Despite the advances in medical diagnostics and broad access to medical facilities, patients with OSCC still have a low 5-year survival rate of around 60% [2].

Therefore, improvements in diagnostic tools involving prevention, early detection, and intraoperative control of resection margins have a valuable role in improving life quality and extending the overall survival rate of OSCC patients. 

The routine histopathological examination includes a conventional investigation of hematoxylin and eosin (H&E) stained specimens utilizing light microscopy. The increasing application of digitalization in histopathology has led to growing interest in machine learning, in particular deep learning and image processing techniques, for a fast and automatized histopathological investigation. 

Ex vivo fluorescent confocal microscopy (FCM) is a novel technology successfully applied for tissue visualization in cellular resolution on the breast, prostate, brain, thyroid, esophagus, stomach, colon, lung, lymph node, cervix, and larynx [3,4,5,6]. Furthermore, our working group recently provided a number of works showing high agreement of confocal images with histopathological sections [7]. Chair-side, bed-side, or intraoperative application is one interesting field of application for this technology. An automated approach could be a major improvement, as it provides a surgeon (with no histopathological background) necessary decision-supportive information fast and effectively. Moreover, deep learning models may help to reduce interobserver biases in the evaluation of tabular data and images [8].

Deep learning utilizing convolutional neural networks (CNNs) is a useful state-of-the-art technique in processing and analysis of a large number of medical images [9]. Numerous studies have already proved the efficiency of computational histopathology applications for automated tissue classification, segmentation, and outcome prediction [10,11,12,13,14,15,16,17,18,19,20,21,22,23]. These investigations provided vast new opportunities to improve the workflow and precision in cancer diagnosis, particularly in primary screening, to aid surgical pathologists and even surgeons.

There are several challenges in the application of deep learning models on pathological slides or confocal scans. For a so-called “supervised learning”, a digital image of at least 20× magnification has to be divided into hundreds to thousands of smaller parts. Each of them has to be annotated manually or semi-manually, which can be extremely time-consuming. The classifier is then applied to each tile to generate the final classification as a sum of all smaller tiles. Alternatively, a weakly supervised learning approach can be applied as the deep learning classifier. A significantly higher number of cases/images is required in this case. There are a number of successful works for both learning models, for example, with a small number of cases (11 images with stomach carcinomas) [24] or with a large dataset with more than 44,000 histological images [23].

Convolutional neural networks (CNNs) are popular machine-learning architectures to process multiple arrays that include the feature information, for example, obtained from images. CNNs often include several layers, such as convolutional and pooling layers, before reaching the fully connected layers and the output layer. While passing architecture, images are abstracted into a feature map from which the information gets processed by the convolutional neurons.

Here, we propose a deep learning model on a limited number of OSCC images obtained with an ex vivo fluorescent confocal microscope and hypothesize that machine learning could be successfully applied to identify relevant structures for cancer diagnosis.

## 2. Materials and Methods

### 2.1. Patient Cohort

From January 2020 to May 2021, a single-center observational cohort study was performed and, prior to investigations, reviewed and accepted by the ethics committee for clinical studies of the Heidelberg University (registry number S-665-2019). The study included (1) patients who gave written informed consent (or their parents if a patient was younger than 18 years); (2) participants were diagnosed as having an OSCC; and (3) resection of the tumor was indicated. The exclusion criteria were: (1) benign pathology of oral mucosa; and (2) scars, previous surgery, or other treatments. Twenty patients with histologically proven OSCC were selected, and a total of 20 specimens were identified, removed, and described clinically and histopathologically (tumor location, histological grading).

### 2.2. Tissue Processing and Ex Vivo FCM

Immediately after resection, the carcinoma excisions were prepared according to the protocol: rinsed in isotonic saline solution, immersed for 20 s in 1 mM acridine orange solution, and rinsed again for approximately 5 s, similar to those already described in other studies [25].

After this, the tissue samples proceeded to ex vivo FCM investigation, which was performed with a Vivascope 2500 Multilaser (Lucid Inc., Rochester, New York, NY, USA) in a combined reflectance and fluorescence mode [25]. The standard imaging of a 2.50 × 2.50 cm tissue sample took, on average, between 40 and 90 s. Hereafter, the samples proceeded to conventional histopathological examinations.

### 2.3. Tissue Annotation

The obtained ex vivo FCM scans of OSCC were annotated according to the WHO criteria: irregular epithelial stratification, the disturbed polarity of the basal cells, disturbed maturational sequence, destruction of the basal membrane and cellular pleomorphism, nuclear hyperchromatism, increase in the nuclear-cytoplasmic ratio, and loss of cellular adhesion and cohesion [26]. Tissue areas that showed clear signs of squamous cell carcinoma were marked using the QuPath bioimaging analysis software. The option to enlarge the tissue samples maintaining high image quality makes a higher precision during the delineation process possible (https://qupath.github.io, accessed on 8 November 2021) [25]. A differentiation between tumor cells and dysplasia was made using different colors. After loading the high-resolution images, the annotation process was conducted. Unclear regions were discussed with an external histopathological specialist. Any regions that did not contain OSCC but included inflammatory or normal tissue were included under the non-neoplastic category. The average annotation time per picture was about 3–4 h. If necessary, the annotations were verified by pathologists. Each annotated image was seen at least by two examiners and one senior histopathologist. Cases with unclear presentations were excluded from training.

### 2.4. Image Pre-Processing and Convolutional Neural Networks

**Figure 1:**
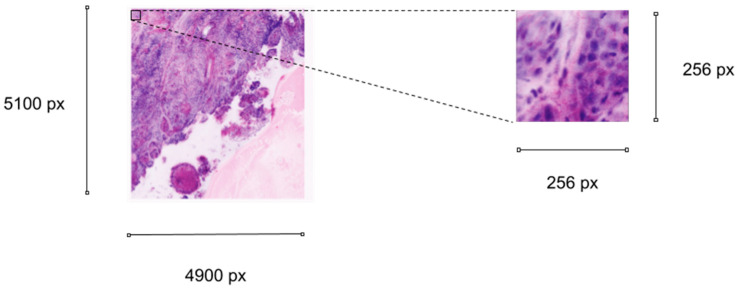
An example of an ex vivo FCM scan with a 256 × 256 px input image.

In most cases, convolutional neural networks consist of multiple layers, including convolutional layers, pooling layers, and fully connected layers. In these cases, the feature extraction in the convolutional layers is followed by pooling and building of one-dimensional vectors for full connection of the layers, resulting in an output layer to solve the classification task. The convolutional neural network used in this study is MobileNet (MobileNets: Efficient Convolutional Neural Networks for Mobile Vision Applications. Available online: https://arxiv.org/pdf/1704.04861.pdf, accessed on 8 November 2021). The main characteristic of this model is the depth-wise separable convolutions, which reduce the processing time as a result of smaller convolutional kernels. We used the Adam optimizer to adjust the weights during the training, and the loss function was the cross-entropy loss. The learning rate was 1 × 10^−5^, and we trained the model for 20 epochs. In order to combat overfitting, additional preprocessing steps were used on the training dataset (i.e., rotation, zooming, and flipping). The input images had a size of 256 × 256 (Figure 1). During the evaluation phase, the trained MobileNet was fed with the entire ex vivo FCM images in a sliding window fashion and generated pixel-level probability maps for both classes.

### 2.5. Training the MobileNet Model

The main principle of a MobileNet model is the application of depthwise separable convolutions. These convolutions separate into a 1 × 1 pointwise convolution with a single filter being applied to each input channel. The pointwise convolution then applies a 1 × 1 convolution to combine the outputs. The depthwise convolution performs a separate application on each channel compared to standard convolutions, where each kernel is applied on all channels of the input image. The MobileNet model was selected due to its satisfying performances in various computer vision tasks and its feasibility towards real-time applications and transfer learnings for limited datasets. Further, this approach significantly reduces the number of hyperparameters and thus the computational power required for the tasks. Furthermore, the MobileNet uses two global hyperparameters, minimizing the need for extensive hyperparameter tuning to obtain an efficient model. The input layer of our models consisted of 256 × 256 × 3 images. After the application of convolutional layers, an 8 × 8 × 512 image output was obtained. Hereafter, a global average pooling was applied, which then resulted in 2 neurons, meaning that there were 512 × 2 weights in addition to 2 biases for each neuron. The output was a binary classification.

**Figure 2:**
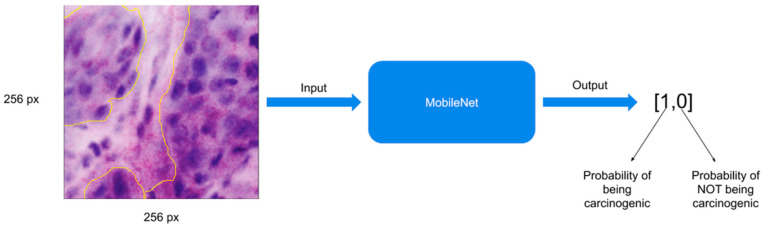
Training a MobileNet: more than 50% of the patch area was annotated as malignant.

For input, if more than 50% of the 256 × 256 pixels area is annotated as cancerogenic, then the whole patch is considered as such and is classified as malignant (Figure 2). From 50,000 generated patches, 8000 were considered as malignant and 42,000 as non-malignant. 

### 2.6. Expanding the MobileNet and Evaluation on the Validation Dataset

For further evaluation, we used the expanded architecture to predict an entire heatmap, which was then compared with the experts’ annotations. Each slide in the validation dataset was split into tiles that were 1024 × 1024 in size. These tiles were sequentially fed to the expanded model, which predicted the heatmap, marking each tile’s most relevant areas. The tiles were then stitched back together in the same order to conserve the location of each tile. The convolutional layers in the expanded architecture resulted in 32 × 32 × 512 output images. Then, the expanded architecture applies an average pooling with “7,7” kernels and “1,1” strides with the same padding to conserve the shape of the input features after the convolutional layers. After forming the fully connected layers, these are transformed into a convolutional layer using the softmax activation function with two filters of size 512. The weights of these filters were the weights from the neurons. The resulting images had a size of 32 × 32 × 2 and were unsampled with a factor of 32 to form the 1024 × 1024 × 2 image output.

**Figure 3:**
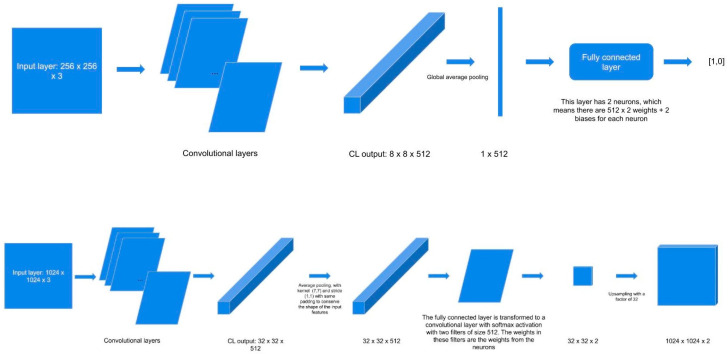
Expanding the MobileNet for evaluation.

Further, post-processing was applied to each heatmap to obtain a segmentation mask (Figure 3):

- Applying a threshold of 0.5 (transform every pixel that has a probability lower than 0.5 to 0 and the rest to 1).
- Erosion (the goal of this operation is to exclude isolated pixels).
- Dilation (after the operation of erosion, the entire mask is slightly thinner, and this operation reverts this property).

The resulting segmentation mask was then compared with the reference to evaluate the performance of the model.

### 2.7. Statistical Analysis

Data analysis was done using cross-validation, where we set the cross-validation parameter k = 10. This split the dataset into 10 groups of 2 cases, and we conducted 10 training sessions, using 9 groups for training and one for validation. The split was unique for each training session. After the training was conducted, an evaluation of the validation dataset was done to calculate the sensitivity and specificity. The training and evaluation were implemented with Python, using Tensorflow and Scikit-Learn.

The following metrics were considered: sensitivity (how much of the truthfully malignant area did get predicted?) and specificity (how much of the truthfully healthy area did get predicted?). All these metrics were computed on a pixel level; each pixel was assigned one of the following labels: true positive (*TP*), the pixel in the predicted mask and the truth mask are both considered malignant; false positive (*FP*), the pixel in the predicted mask is considered malignant, but the truth mask marks it as healthy; true negative (*TN*), the pixel in the predicted mask and the truth mask are both considered healthy tissue; and false negative (*FN*), the pixel in the predicted mask is considered healthy, but the truth mask marks are malignant. Using this setup, the metrics were calculated using the following formulas:

$$Specificity=\frac{TN}{TN+FP}$$

$$Sensitivity=\frac{TP}{TP+FN}$$

## 3. Results

**Figure 4:**
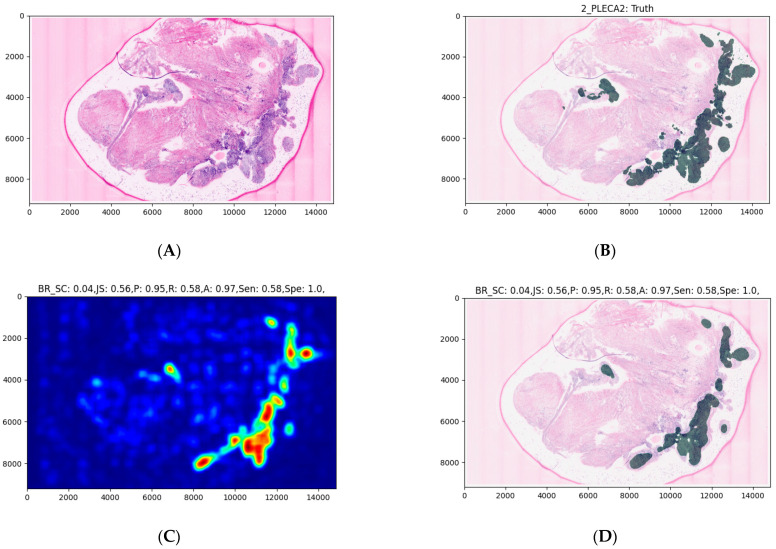

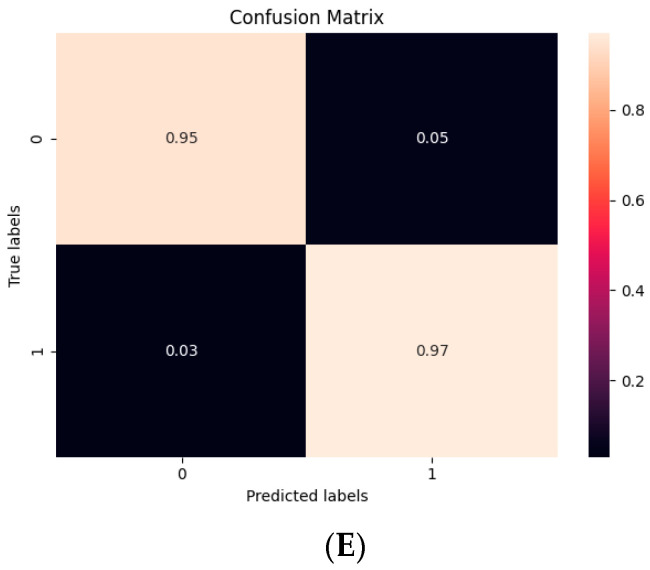
An example of an OSCC ex vivo FCM image with manually annotated cancerous regions and automated prediction. Image (**A**) is the input to the model, i.e., digitally stained ex vivo confocal microscopy scan, which was delivered in a sequential manner as explained in the Section 2. Image (**B**) contains the expert annotation highlighted in green. Image (**C**) shows the predicted heatmap. The colormap used is called jet, and it ranges from blue to red, where the former represents the not SCC and the latter SCC. Image (**D**) presents the prediction mask generated from the heatmap, using the described approach in the Section 2. Image (**E**) is a per-row normalized confusion matrix, the number 0 represents the class, not SCC, and 1 represents SCC. The abbreviations above the images (**C**,**D**) are the following: BR_SC for Brier score, JS for Jaccard score, P for precision R for recall, A for accuracy, Sen for sensitivity, and Spe for specificity. An entire heatmap and segmentation mask was compared with the experts’ annotations. As we can see in this example, the annotated area could be sufficiently predicted by the model.

**Figure 5:**
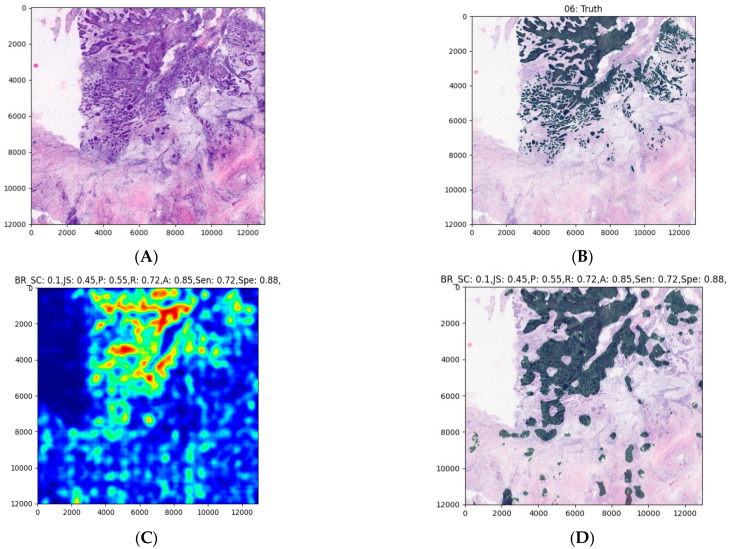

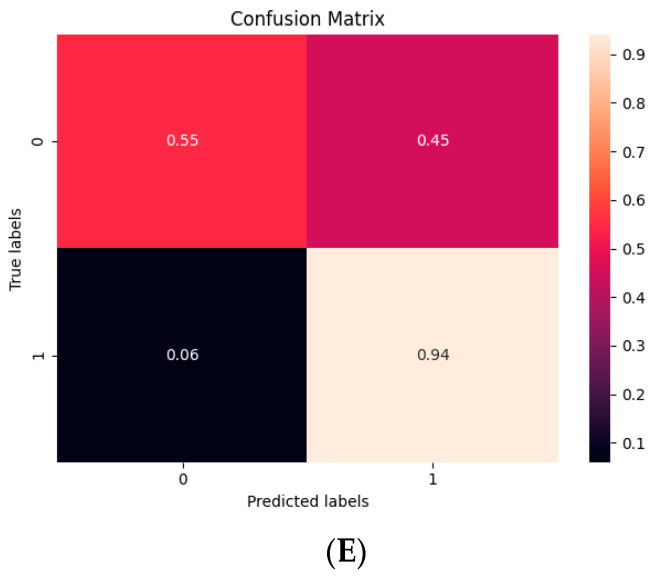
An example of an OSCC ex vivo FCM image with manually annotated cancerous regions and automated prediction. The interpretation of images (**A**–**E**) is the same as in Figure 4. An entire heatmap and segmentation mask was compared with the experts’ annotations. We can observe a less exact recognition of smaller annotations.

**Figure 6:**
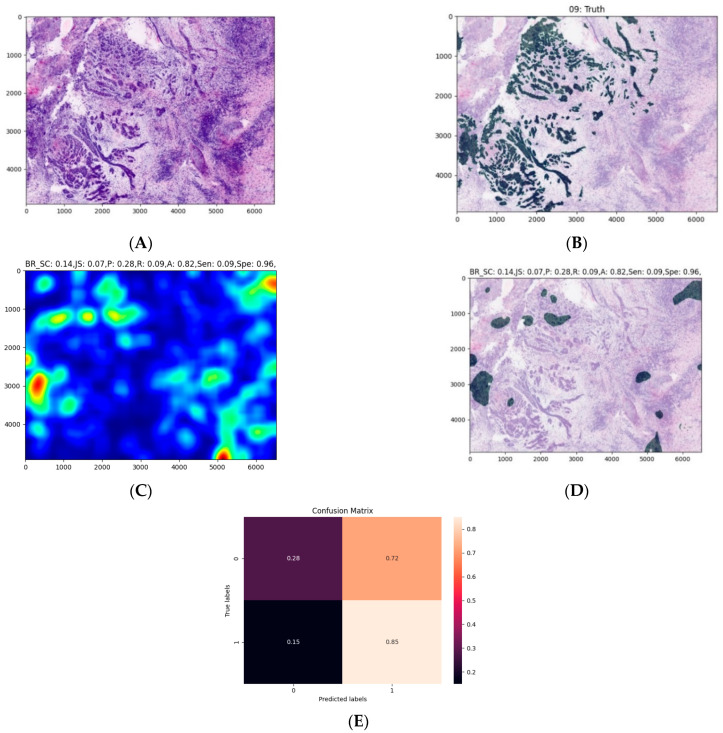
An example of an OSCC ex vivo FCM image with manually annotated cancerous regions and automated prediction. The interpretation of images (**A**–**E**) is the same as in Figure 4. An entire heatmap and segmentation mask was compared with the experts’ annotations. We can observe poor recognition of fragmented annotations.

The average processing time of the model was about 30 s for the largest images and was generally very dependent on the image size. The model seems to correctly highlight larger areas but struggles to find smaller malignant areas (Figure 4, Figure 5 and Figure 6). The model also struggles to identify malignant areas that are very fragmented. The images represent the tissue samples after scanning with ex vivo FCM (A in Figure 4, Figure 5 and Figure 6), annotations of OSCC regions on the same scan (B in Figure 4, Figure 5 and Figure 6), a heatmap (C in Figure 4, Figure 5 and Figure 6), and the area predicted by a model (D in Figure 4, Figure 5 and Figure 6). The digital staining of OSCC regions in pictures B and D is dark green for better contrast and comparability.

The specificity/sensitivity for correct detection and diagnosis of cancerous regions in FCM images achieved 0.96 and 0.47, respectively (Table 1).

## 4. Discussion

This preliminary study aimed to evaluate whether a well-annotated dataset of OSCC samples fed into a convolutional neural network can be successfully used as a cancer tissue classifier. In the current work, we applied a machine learning model on ex vivo confocal images of OSCC. This model was trained on cancerous tissue obtained and evaluated from a single institutional department. The output of the model was a set of heatmaps representing the locations of cancerous and non-cancerous regions for tissue classification. We think that the results of this study are important as, to the best of our knowledge, this is the first study to validate a machine learning model on images of oral squamous cell carcinoma obtained with a novel device different from classic histopathology with an ex vivo fluorescent confocal microscope.

In our model, even though we used a small number of images (*n* = 20), we observed excellent results in terms of specificity (0.96), translating to a high detection rate for healthy non-cancerous regions. In contrast, the sensitivity of the described method only achieved 0.47. This is somewhat to be expected as a result of architectural heterogeneity in cancerous regions (different nuclear polarization, size, and orientation of cells). The healthy tissue regions represent graphically clear and similar patterns on a cellular level. Thus, a higher number of images and data material might be supportive of reaching a high sensitivity in automated detection of cancerous areas.

This also serves as the main limitation of the present study: a larger amount of data would be required for reliable results in the automated classification of OSCC. Especially multi-center studies involving large cohorts are warranted, as in single institutional cohorts, the collected samples may not represent the true diversity of histological entities. In addition, in our study, we applied just one CNN model to verify our results; verification of results with other CNN architecture would be necessary to determine the best architecture for the respective dataset. In general, a successful predictive model requires a large amount of data for sufficient CNN training [27]. In oncologic pathology, the problem has been or is currently being solved through multicenter collaboration and establishment of databases to cover all rare cases on the field. Due to the novelty of applied technology, there is not much data with ex vivo FCM images and almost no material in oral pathology. However, since the digitalization of pathological images and the invention of whole slide images (WSIs) with gigapixel resolution, it has become possible to analyze each pixel and enhance the data volume [12,28].

At the beginning of the computational pathology era, the main works were focused on traditional pathology methods of mitosis counting [9,29,30]. Since the process was extremely time-consuming and investigator dependent, new diagnostic features for computational malignancy identification had to be investigated. For example, in 2014, a working group [31] achieved high accuracy in distinguishing not only between benign breast ductal hyperplasia and malignant ductal carcinoma in situ but also between the low-grade and high-grade variants. This was possible through the analysis of nuclear size, shape, intensity, and texture.

Concerning oral oncology, there are just a very few studies, and most of them are focused on prognostication and survival and recurrence prediction in OSCC patients [32,33,34,35,36,37,38]. These works use machine learning to analyze demographic, clinical, pathological, and genetic data to develop a prognostic model. One working group [39] developed a model for OSCC prognosis in 2013 with the AUC of 0.90 based on p63, tumor invasion, and alcohol consumption anamnesis.

A couple of studies investigated the possibilities of automated detection of OSCC regions using optical imaging systems. For example, an accuracy of 95% for OSCC classification was achieved in the study by Jeyaraj et al. [40].

Machine learning applied on digital histopathological images of OSCC was described by Lu et al. [41], where each tissue microarray was divided into smaller areas and analyzed as a disease survival predictor. The specificity and sensitivity achieved with this classifier (71% and 62%, respectively) showed promising results for the investigation of small areas of tissue.

Evaluation of oral cancer detection utilizing artificial intelligence has been performed by testing numerous machine learning techniques in the past. Principal component analysis and Fisher’s discriminant are often used to build promising algorithms. There is evidence that support vector machine, wavelet transform, and maximum representation and discriminant feature are particularly suitable for solving such binary classification problems. For example, in early research of Krishnan et al. focusing on oral-submucosal fibrosis, a support vector machine for automated detection of the pathology was able to reach an accuracy of 92% [42]. In addition, Chodrowski et al. applied four common classifies (Fisher’s linear discriminant, kNN-Nearest Neighbor, Gaussian quadratic, and Multilayer Perceptron) to classify pathological and non-pathological true color images of mouth lesions, such as lichenoid lesions. They reported that the linear discriminant function led to the most promising accuracy results, reaching nearly 95% considering 5-fold cross-validation [43]. Although this study reveals the potential of intraoral pathology detection by combining optical imaging and machine learning techniques, the intraoperative validation of wound margins would not be possible with this approach. The MobileNet model used by us was selected due to its resource efficiency, the promising available evidence on skin lesion detection [44,45], and its feasibility for real-time applications, making it exceedingly feasible for the combination with ex vivo confocal microscopy. The depthwise separable convolutions allowed us to build lightweight deep neural networks, and we applied two simple global hyper-parameters that efficiently reduce the required computational resources. Further, the model is suitable to assess limited datasets and minimizes the need for extensive hyperparameter tuning while evaluating the model in the preclinical stage. Notably, a multi-class detection model combining optical imaging and machine learning to solve classification problems is highly warranted in the future and would improve the method presented here. Valuable data regarding this idea was provided recently by Das et al. [46]. The authors assessed multiple machine learning models for automated multi-class classification of OSCC tissues. Their model could be of high interest in the future when combined with ex vivo fluorescence confocal microscopy in the way our workgroup applied it. Consequently, the affected tissue can not only be classified as pathological or not (binary classification) but also subtypes of OSCC or gradings can be considered. Further, they provided important comparisons of different machine learning models that will help to find the most promising combination of technical application (optical imaging) and neural network model to solve OSCC classification problems. Based on our findings and the evidence available in the literature, this will be of high clinical relevance in the future.

## 5. Conclusions

Given the early promising results in the field of OSCC detection by optical imaging, we showed the feasibility of a novel diagnostic approach compared to classic histopathology in the current study. We encourage other workgroups to do more research on deep learning methods in cancer medicine to promote the current developments of precise, fast, and automatized cancer diagnostics.

## Figures and Tables

**Table 1:** Sensitivity and specificity of OSCC diagnosis utilizing the MobileNet model.

	10 Fold (*n* = 20, t = 0.3)
Sensitivity	0.47
Specificity	0.96

We performed 10-fold cross-validation, measured the respective metrics for every case in the validation set for every fold, and reported the average overall fold. *n* = number of samples, t = classification threshold used to convert the heatmap into a segmentation mask.

## Data Availability

The data presented in this study are available to each qualified specialist on reasonable request from the corresponding author. The whole datasets are not publicly available as they contain patient information.
